# Clonal Complexes 23, 10, 131 and 38 as Genetic Markers of the Environmental Spread of Extended-Spectrum β-Lactamase (ESBL)-Producing *E. coli*

**DOI:** 10.3390/antibiotics11111465

**Published:** 2022-10-24

**Authors:** Lara Pérez-Etayo, David González, Ana Isabel Vitas

**Affiliations:** 1 Department of Microbiology and Parasitology, University of Navarra, 31008 Pamplona, Spain; 2 Navarra Institute for Health Research (IdiSNA), 31008 Pamplona, Spain

**Keywords:** antimicrobial resistance, CCs, phylogroups, *One Health*, STs

## Abstract

In accordance with the global action plan on antimicrobial resistance adopted by the World Health Assembly in 2015, there is a need to develop surveillance programs for antimicrobial resistant bacteria. In this context, we have analyzed the clonal diversity of Extended-spectrum β-lactamase (ESBL)-producing *Escherichia coli* (*E. coli*) isolated from aquatic environments and human and food samples in Spain, with the aim of determining possible clonal complexes (CCs) that act as markers of the potential risk of transmission of these resistant bacteria. The phylogenetic groups, sequence types (STs) and CCs were determined by different Polymerase Chain Reaction (PCR) and Multilocus Sequence Typing (MLST) techniques. Phylogroup A was prevalent and was mainly present in food and water strains, while human strains were mostly associated with phylogroup B2. According to the observed prevalence in the different niches, CC23 and CC10 are proposed as markers of phylogroups A and C, related with the spread of *bla*_CTX-M1_ and *bla*_CTX-M15_ genes. Similarly, CC131 and CC38 could be associated to the dissemination of pathogenic strains (phylogroups B2 and D) carrying mainly *bla*_CTX-M14_ and *bla*_CTX-M15_ genes. Some strains isolated from wastewater treatment plants (WWTPs) showed identical profiles to those isolated from other environments, highlighting the importance that water acquires in the dissemination of bacterial resistance. In conclusion, the detection of these genetic markers in different environments could be considered as an alert in the spread of ESBL.

## 1. Introduction

Bacterial antimicrobial resistance (AMR) has emerged as one of the main public health threats of the 21st century [1]. The list of global priority pathogens resistant to antibiotics includes enterobacteria resistant to 3rd generation of cephalosporins among the 12 families of bacteria most dangerous to human health [2], being *Escherichia coli* (*E. coli*) one of the major extended-spectrum β-lactamase enzymes (ESBL)-producer. The World Health Organization (WHO) points out the need to address the problem of antimicrobial resistance from a global approach (in accordance with the *One Health* initiative) and promotes a series of strategies to contain the spread of the resistances [3], including the surveillance of resistant bacteria in different environments [4].

*E. coli* is a very ubiquitous Gram-negative micro-organism [5] that, in addition to being part of the intestinal microbiota of most animal species [6], is one of the main etiological agents of different urinary or gastrointestinal infections [7]. The great diversity of *E. coli* strains has given rise to various classifications over the years [8]. According to their pathogenic capacity, commensal intestinal and extraintestinal strains can be differentiated, and it has been seen that they harbor numerous virulence factors and antibiotic resistance genes [9]. Herzer et al. [10] conceived a classification system in four phylogroups (A, B1, B2 and D) with independence of the pathogenic capacity. However, a relationship was later found between the different phylogroups and the virulence of the strains [11]. Thus, phylogroups A and B1 harbor commensal strains with low virulent power, while phylogroups B2 and D include mainly extraintestinal pathogenic strains that express virulence factors responsible for promoting the stages of colonization, adherence, invasion, etc. [12,13].

Afterward, Clermont et al. [14] developed a molecular technique for the detection of these phylogenetic groups, which was improved years later by modifying the primers and the Polymerase Chain Reaction (PCR) conditions [15]. Thus, the new phylogenetic classification contains seven phylogroups (A, B1, B2, C, D, E and F) that belong to the species *E. coli sensu stricto* and one xmore that belongs to the *Escherichia* clade [16]. This clade is divided into five groups (Clades I-V), phenotypically and biochemically very similar to *E. coli* but genetically distant [17]. Given the interest in phylogenetic studies of these strains, Wirth et al. [18] developed a database for the study of *E. coli* using Multilocus Sequence Typing (MLST). Thus, each strain of *E. coli* is associated with a sequence type (ST) and clonal complexes (CC) and various studies have shown the great diversity of existing STs among the different ESBL-producing *E. coli* isolates. In addition, multiple associations have been found between different STs and phylogroups, as ST131 and phylogroup B2 [19,20].

Thus, the main objective of this work was to determine possible CCs that could act as markers of the potential risk of transmission of β-lactam antibiotic resistances. The study was carried out in ESBL-producing *E. coli* strains isolated by our research group from diverse sources in the north of Spain (Navarra) in previous studies [21,22,23,24]. The sources were selected according to the observed high prevalence of these resistant strains and/or the presence of clinically relevant resistant bacteria. This way, the phylogenetic relationship among ESBL-producing *E. coli* isolated from foods (chicken and turkey meat), human (healthy people) and aquatic environments (rivers, collectors and wastewater treatment plants [WWTP]) was determined.

## 2. Results

### 2.1. Classification of E. coli ESBL Strains According to Phylogenetic Groups

The 61 *E. coli* strains were classified into the different phylogenetic groups mentioned above: phylogroup A being the largest (29.5%), followed by B1 (19.7%) and B2 (14.8). On the contrary, phylogenetic groups C, E and Clade I or II were the least prevalent (4.9%, 1.6% and 1.6%, respectively). On the other hand, in 8.2% of the strains (*n* = 5) no determining results of phylogenetic classification were obtained, so it was necessary to perform the MLST technique.

**Figure 1:**
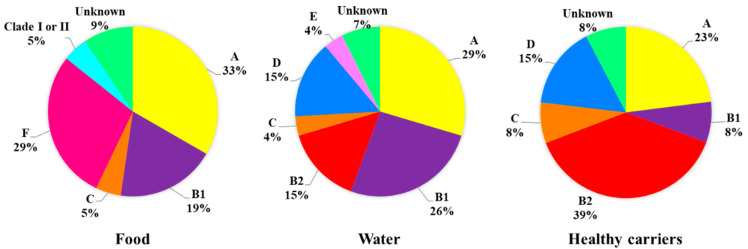
Distribution of *E. coli* strains in phylogenetic groups according to their origin.

A heterogenous distribution of phylogroups was observed according to the origin of strains (Figure 1). Thus, strains isolated from food and water, mainly belong to phylogroup A, while human strains (healthy carriers) are mostly associated with phylogroup B2. In this sense, significant differences (*p* < 0.005) were found in the frequency of phylogroup B2 between food and water samples (*p* = 0.0372) and between food and healthy carriers (*p* = 0.0068).

In this way, phylogroups B2 and D, which mainly include extraintestinal pathogenic strains, were identified only among strains isolated from water and from healthy carriers, with a higher prevalence of group B2 within the human strains. On the contrary, phylogroups A, B1 and C (mainly harboring commensal strains with low virulent power) were detected in the three environments studied, with no significant differences in the observed prevalence (*p* > 0.05) (in the case of phylogroup B1 probably due to the small sample size of healthy carriers). On the other hand, it should be mentioned that the detection of the new phylogroup E in a single strain isolated from water and the fact that phylogroups F and Clade I or II were only present in food strains (no significant differences).

**Figure 2:**
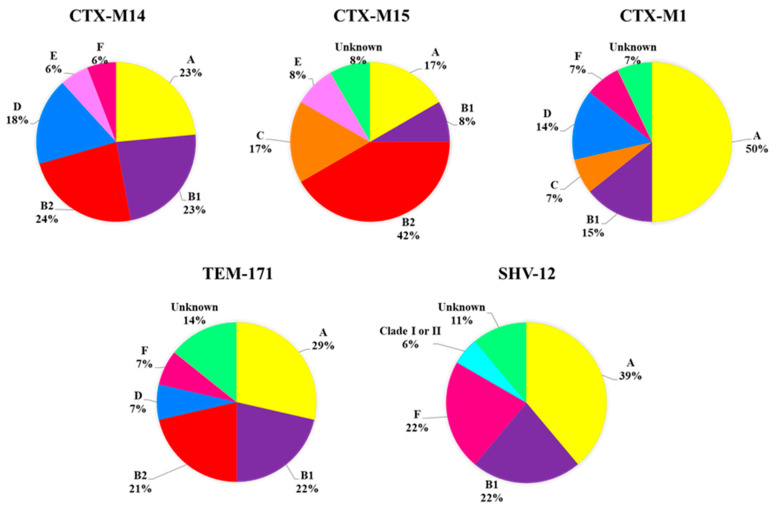
Distribution of *E. coli* strains producing different types of ESBLs within the phylogenetic groups.

At the same time, the relationship of the main *bla* genes present in the 57 strains, with the phylogenetic groups was also studied (Figure 2), not finding significant differences between any of these percentages (*p* > 0.05). Despite no substantial association of *bla* genes with a certain phylogenetic group being found, *E. coli* strains producing *bla*_CTX-M15_ belonged mostly to phylogroup B2 (42%) and *bla*_CTX-M1_ to phylogroup A (50%). Phylogroups A, B1, F, and D were found to be associated with all ESBL types (except CTX-M15 in the case of phylogroups F and D; and SHV-12 in phylogroup D). On the other hand, phylogenetic group B2 was associated with genes *bla*_CTX-M14_, *bla*_CTX-M15_ and *bla*_TEM-171_, while group C was only found in association with *bla* genes of the CTX-M type (except *bla*_CTX-M14_). Finally, it should be noted that *Clade* I or II was only detected in strains carrying *bla*_SHV-12_ and phylogroup E was only associated with a strain carrying two types of β-lactamases (CTX-M14 and CTX-M15).

### 2.2. Analysis of CCs and STs by MLST

**Figure 3:**
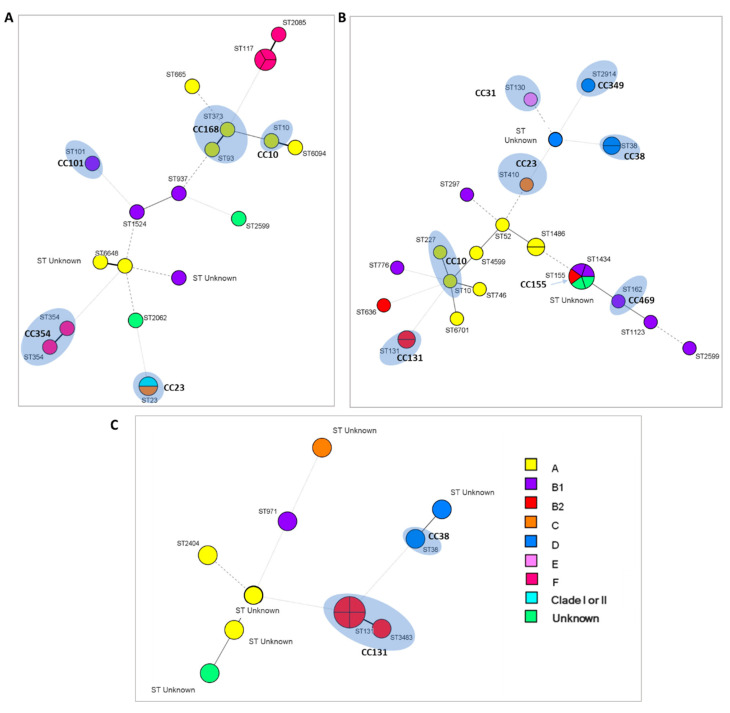
Clonal composition by MST of *E. coli* strains isolated from (**A**) food, (**B**) water and (**C**) healthy carriers. Each circle corresponds to an ST, its size being proportional to the number of isolates. The color of the circles indicates the phylogroup to which each isolate belongs, as indicated in the legend. The shaded areas indicate the detected CCs that group different STs. The union lines between the different nodes (bold, continuous, dashed or dotted lines) show the number of discordant alleles between the profiles of the strains (1, 2, 3 and 4 alleles, respectively).

**Figure 4:**
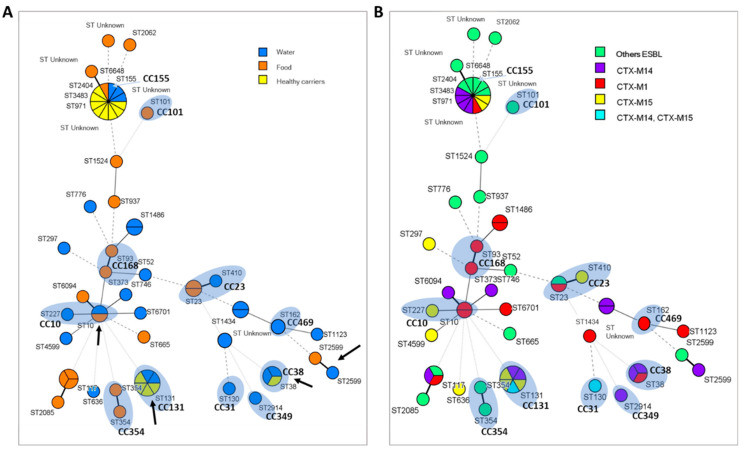
Clonal composition by MST of the 61 *E. coli* strains analyzed according to the source of isolation (**A**) and in relation to the type of β-lactamase produced (**B**). Each circle corresponds to an ST; its size being proportional to the number of isolates. The shaded areas indicate the detected CCs that group different STs. The union lines between the different nodes (bold, continuous, dashed or dotted lines) show the number of discordant alleles between the profiles of the strains (1, 2, 3 and 4 alleles, respectively) and the arrows indicate the same *ST* that appear in different niches.

The analysis of the data obtained by MLST allowed us to establish the taxonomic and phylogenetic relationships between the strains by creating different phylogenetic trees, as shown in Figure 3 and Figure 4.

Regarding the strains isolated from turkey and chicken meat, the analysis showed a great diversity of STs. A total of 15 different STs was detected among 21 strains (Figure 3A), being the prevalent ST117 (14.3%), followed by ST23 (9.5%) and ST354 (9.5%). In terms of CCs, 5 different were detected. Because to date ST117 is not associated with any known complex, the predominant were CC23, CC354 and CC168 (9.5%), followed by CC10 and CC101. In addition, despite the distances found in the phylogenetic tree, the three strains belonging to ST117, the strain ST2085 and the two strains ST354, belong to the new phylogroup F. The strains grouped in the CC168 and CC10 were associated with phylogroup A, while the two belonging to CC23 were from different phylogroups (C and Clade I or II).

In relation to the strains isolated from aquatic environments (Figure 3B), the results showed the presence of 20 different STs in 27 strains, ST131, ST1434, ST1486 and ST38 (7.4%) being the most frequent, while 3 of the strains were not associated with any known ST. Eight different CCs were detected, with an identical prevalence of CC10, CC131 and CC38 (7.4%). It should be noted that all of them have been detected in water from WWTP or slaughterhouse collector, with the exception of CC23 that has been detected in a river (Table S1). As described in the literature [19,20], the two strains associated with the CC131 were included within the pathogenic phylogroup B2, while the strains of the CC38 were associated with the phylogroup D. On the other hand, the strain carrying the *mcr-1* colistin resistance gene was assigned to phylogroup B2, despite the determined ST155 being phylogenetically closer to the ST1434 (phylogroup B1). In general, phylogroups A and B1 were associated with a higher diversity of STs, which were spread throughout the phylogenetic tree. CC10 (related to phylogroup A) was one of the previous strains isolated from chicken samples. The same happened with the CC23, which was detected in only one strain and in this case belongs to phylogroup C. Finally, the new phylogroup E detected in a single strain seems to be related to the CC31 (ST130).

Finally, the clonal composition of the 13 ESBL-producing *E. coli* strains isolated from healthy carriers is shown in Figure 3C. Only five ST were determined, being ST131 the most prevalent (30.8%), while the remaining five strains were not related to any known ST. At the same time, two different CCs were established and, as can be seen in the Minimum Spanning Tree (MST), both complexes are close and related with the more pathogenic phylogroups. The four strains corresponding to ST131 and ST3483 (all of them belonging to phylogroup B2) were defined as CC131, while the CC38 included only strain ST38, associated with phylogroup D.

#### ST and CC Relationship of Strains Isolated from Different Environments

In total, 36 STs and 11 CCs have been identified in this study and none of them has been present in the 3 environments (Figure 4A). However, it can be seen that despite not finding a common CC, there were strains phylogenetically closer (huge circle) that were isolated in the three environments. Most of these strains were ESBL producers and in addition, three of them were resistant to colistin (Table S1). Likewise, the CC131, CC38, CC10, CC23 and the corresponding ST (indicated with arrows in the Figure 4A) appear in two different environments. In this sense, the CC131 (associated with phylogroup B2) was present in four strains from healthy carriers and two strains from aquatic environments. The same occurred with the CC38 (associated with phylogroup D), disseminated in the aforementioned environments. On the contrary, the CC10 and CC23 were present in strains from food and aquatic environments. It should be noted that there has been no coincidence between strains isolated from food and healthy carriers, probably because bacterial inactivation occurs during cooking of chicken and turkey meat.

At the same time, a study of the different STs and CCs was also carried out in relation to the type of β-lactamase produced by each strain (Figure 4B). Phylogenetic relationships have been established taking into account the CTX-M group as the main *bla* gene when the strains expressed more than one different type of β-lactamase. In the case of presenting only another type of gene (*bla*_SHV_ or *bla*_TEM_), these are shown in the phylogenetic tree as “other ESBLs” as indicated in the legend. The 6 strains belonging to the CC131 (isolated from healthy carriers and water) are producers of CTX-M14 and CTX-M15 (or even both β-lactamases together), while the CC38 detected in these same environments is related with strains carrying *bla*_CTX-M14_ and *bla*_CTX-M1_. In the case of strains isolated from water and food belonging to CC10, these are producers of CTX-M1 and CTX-M15, the same as strains belonging to CC23, which also includes a producer of SHV-12. On the other hand, the strains grouped in the CC168 are carriers of *bla*_CTX-M1_, unlike those associated with CC354 are producers of other types of β-lactamases, such as SHV-12 or TEM-171. In the same way, the strains corresponding to the CC101, as well as other STs (ST117, ST1524, ST937, etc.) are also producers of these types of β-lactamases. Finally, the corresponding strains with the ST971, ST746, ST2404 and ST6094, as well as other unknown ones, are producers of CTX-M14.

## 3. Discussion

The data obtained in this study show the phylogenetic relationships between ESBL-producing *E. coli* strains isolated from different out-of-hospital environments (animal source foods, aquatic environments and healthy people) in Navarra, northern Spain.

The prevalent phylogenetic groups in the studied foods have been A (33%), F (29%) and B1 (19%), very similar to that observed in strains isolated from water with the exception of phylogroup F, which has not been detected (29% and 26%, groups A and B1, respectively). By contrast, the prevalent phylogroup in healthy carriers was B2 (39%), followed by groups A (23%) and D (15%). A relationship between virulence and phylogeny in *E. coli* strains has been established based in most cases on the study of the 4 main phylogenetic groups (A, B1, B2 and D) [25,26]. In this way, the strains that cause extraintestinal infections have been associated with phylogroup B2 and to a lesser extent with D, while the commensal strains are associated with groups A and B1 [27]. However, according to the latest updated scheme by Clermont et al. [15], the groups are restructured as follows: phylogenetic group A is reclassified into A and C; the phylogenetic group B2 would include B2 and E, while D can be divided into D and F. Although this new classification was published in 2013, there are still few studies based on it, so there is not much information about the characteristics and distribution of the new phylogroups, C, E, and F, and the cryptic clades of *Escherichia* among the different ecosystems. In this line, our results coincide with those published by Coura et al. [28] who reported the prevalence of groups related to commensal strains (A, B1 and C) among 149 poultry isolates. Similarly, Tansawai et al. [29] determined that the majority of ESBL-producing *E. coli* isolated from poultry meat (chicken, duck and poultry) belonged to phylogenetic group A (51%) and B1 (27.5%), reinforcing the idea of the association of phylogroups A, B1 and C with commensal strains. In our study we have detected these three phylogroups in all the environments studied, with phylogroups A and B1 being the most frequently detected in samples from foods of animal origin, as described by Carlos et al. [26]. On the other hand, the presence of the new phylogroup F in strains isolated from processed chicken products should be mentioned, since this phylogroup has recently awaked interest due to its association with extraintestinal pathogenic *E. coli* (ExPEC) strains in humans [30,31] and domestic animals [32,33]. However, its lacks the virulence characteristics typically associated with phylogroup B2 causing extraintestinal infections [31]. On the contrary, the most pathogenic groups B2 and D have been detected both in aquatic environments and in healthy carriers, with a high prevalence of phylogroup B2 in the latter (39%). Both phylogroups have been associated with urinary tract infections and bacteremia in immunosuppressed patients [34]. These data are consistent with other studies [20,35], which also associate these phylogenetic groups with strains carrying *bla*_CTX-M15_, coinciding with our results (Figure 4). In addition, phylogroup D was also detected in strains from wastewater and rivers in previous studies carried out by our research group [36]. On the other hand, *E. coli* strains belonging to the phylogenetic group E have been reported to be more prevalent in the gastrointestinal tract of wild mammals than in humans [15,27,37], which is consistent with our results, since this phylogroup has only been detected in aquatic environments, with which wild mammals have been able to have close contact. At the same time, 5% of food isolates were classified as cryptic clades of *Escherichia*, that is, biochemically and phenotypically identical strains of *E. coli* but genetically distant. The cryptic clades of *Escherichia* were defined in 2009 by Walk et al. [38] and although there are very few studies in this regard, they seem to correspond to strains better adapted to the natural environment (especially Clades II-IV), such as water, soils or sediments [16]. Thus, animals could act as occasional reservoir of this phylogroup due to their close contact with the natural environment. Most cryptic *Escherichia* strains are characterized by lower levels of resistance and virulence (except Clade I), but there are few studies indicating the differences in resistance patterns between the clades [17,39]. In our case, the data obtained in the study of resistance patterns (MicroScan), reveal resistance to penicillins, cephalosporins, monobactams, tetracyclines and some type of quinolones, in addition to being a carrier of the *bla*_SHV-12_ gene (Table S1, strain 10). Due to the distinction between the clades is based on biochemical markers, further studies would be necessary to characterize whether this strain corresponds to Clade I or II.

MLST studies revealed the presence of multiple STs and 11 well-defined CCs among the strains tested. No CCs were found that brings together strains from the 3 environments, however, 4 CCs associated strains from 2 niches, so they could act as genetic markers for the surveillance of β-lactam antibiotics resistance. Specifically, CC23 and CC10 were detected in samples of water and food, while CC131 and CC38 were present in aquatic environments and healthy carriers. In this sense, the presence of the CC10 (associated with phylogroup A) in chicken and water stands out, coinciding with the results published by other authors who determined the spread of this CC in food and environmental settings [40,41,42]. Although this CC is commonly associated with strains of low virulent power, it has been related to human infections and ESBL-producing strains with variants belonging to the *bla*_CTX-M1_, *bla*_CTX-M2_ and *bla*_CTX-M9_ groups in different ecosystems [43]. In our study, these three *E. coli* CC10 strains are carriers of *bla*_SHV-12_, *bla*_TEM-171_, *bla*_CTX-M1_ and *bla*_CTX-M15_, and in addition to being resistant to β-lactam antibiotics, they are also resistant to other families of antibiotics (Table S1). On the other hand, CC23, which has been well characterized and is disseminated throughout various environments, as shown by other researchers [44,45], has been detected in strains isolated from food and water: two belonging to the phylogroup C-*bla*_CTX-M_ and another to Clade I or II-*bla*_SHV-12_. One of the most prevalent CC has been CC131 (11.5% *n* = 6), associated with more virulent strains related to phylogroup B2 [19,46] and which has become one of the most important pandemic clones in the rapid global spread of the *bla*_CTX-M15_ genes and to a lesser extent *bla*_CTX-M14_ [5,47]. As can be seen in Figure 4, all strains associated with this complex have been isolated from healthy carriers and aquatic environments and are producers of CTX-M14 y CTX-M15 enzymes. Finally, another CC detected in these same environments has been CC38 (5%), which comprises important lineages related to the dissemination of ESBL/AmpC genes between the human, livestock and environmental environments [48]. In this same line, the strains associated with this complex were related to phylogroup D, belonging to virulent strains and were carriers of *bla*_TEM-171_, *bla*_CTX-M14_ and *bla*_CTX-M1_ genes.

Regarding others ST detected in rivers, ST1434 was associated with phylogroup B1 and with the spread of *bla*_CTX-M14_ genes. Similarly, the association of this ST with resistance to β-lactams and carbapenems was previously described by Piedra-Carrasco et al. [49] in other rivers in northern Spain. In the case of ST1486, in this work it has been related to the commensal phylogroup A and *bla*_CTX-M1_ genes, meanwhile Zhang et al. [50] had observed the presence of this ST in retail pork isolates, that were also carriers of NDM-5 carbapenemases. On the other hand, the colistin-resistant strain number 48 (positive for the *mcr-1* gene and isolated from the collector of a rabbit slaughterhouse, Table S1), has been found to belong to the CC155 and phylogroup B2. These results are in agreement with those presented by Jamborova et al. [51], who determined that the strains associated with this complex have been frequently related to infections in livestock in Europe. In relation with food isolates, ST117 was the most prevalent. Although to date this ST is not associated with any known CC, it is well characterized as the cause of avian colibacillosis (APEC “avian pathogenic *E. coli*”) [52]. This is consistent with our results, since all the ST117 strains associated with the phylogroup F and carrying the *bla*_CTX-M1_, *bla*_CTX-M14_ and *bla*_SHV-12_ genes have been isolated from chicken samples, in a similar way to that described by Ben Said et al. [44]. ST117 is a potential zoonotic that poses a risk of extra-intestinal infections in humans [53], therefore surveillance of these strains raises concerns [54]. However, there is a lack of understanding of virulence traits, antimicrobial resistance patterns, and genomic evolution [55]. Likewise, the CC168 (associated with phylogroup A) has been detected in two strain carriers of *bla*_CTX-M1_ and *bla*_SHV-12_ isolated from food, consistent with previous studies in the same region [36]. The CC354 has been observed in two food strains associated with phylogroup F and, as in the work of Guo et al. [33], both are resistant to quinolones. This fact is relevant since this resistance is closely related to the use of quinolones in poultry [56], especially in chickens, the source of most of the analyzed food samples.

In summary, the large number of phylogroups, STs and CCs shows the great genetic diversity that exists between *E. coli* populations. However, strains isolated from WWTPs have shown identical profiles to those of strains isolated from other environments, highlighting the importance that water acquires in the dissemination of bacterial resistance. According to the obtained results, CCs 10 and 23 are proposed as markers of phylogroups A and C, respectively. Their detection in samples from animal and food environment could be considered as a possible warning of the spread of β-lactam resistance, due to the relationship with *bla*_CTX-M1_ and *bla*_CTX-M15_ genes. On the other hand, clones CC131 and CC38 could be used as markers with great clinical relevance, based on their relationship with pathogenic strains belonging to phylogroups B2 and D, respectively, as well as their association with *bla*_CTX-M14_ and *bla*_CTX-M15_ genes. In the framework of the *One Health* initiative, surveillance programs should include the monitoring of different sources, with especial emphasis in aquatic environments to detect ESBLs dissemination between environment, animals and people.

## 4. Materials and Methods

### 4.1. Selection of E. coli Strains

A total of 59 *E. coli* strains isolated from processed turkey and chicken foods (*n* = 21), water (*n* = 25) and healthy carriers (*n* = 13), were selected from a collection of strain isolated in previous studies. Previous phenotypic characterization confirmed that they were ESBL producers, although β-lactamase genes were not detected in two of them [22,23,24]. In addition, 2 colistin-resistant strains isolated from water were included in the study (given the relevance of resistance to this antibiotic used in the treatment of infections caused by multiresistant bacteria), with the presence of the *mcr-1* gene in one of them [23]. The main characteristics of the 61 strains in relation to the source, resistance genes and the resistance profile to different antibiotics are shown in the supplementary material (Table S1). It should be noted that 96.7% of the strains had a multi-drug resistance (MDR) profile and 73.8% extended MDR (resistance to at least three or five families of antibiotics, respectively).

### 4.2. Bacterial DNA Extraction

From fresh cultures of each strains in Triptic Soy Agar (TSA, Scharlab, Barcelona, Spain), 5 mL of Brain Heart Infusion broth (BHI, Scharlab, Barcelona, Spain) were inoculated and incubated at 37 °C for 18–24 h. From this, 2 mL were centrifugated at 5000× *g*. for 10 min (Fisher MicroCentrifuge Model 235A) and the total DNA was extracted using the DNeasy^®^ Blood & Tissue kit (Qiagen, Barcelona, Spain), with a modified pre-treatment protocol for Gram-negative bacteria, following the manufacturer’s instructions. Finally, the quantity and quality of the DNA was analyzed using the ND-1000 spectrophotometer (NanoDrop Technologies, Wilmington, DE, USA). The extracts were stored at −20 °C until use.

### 4.3. Determination of Phylogenetic Groups

The determination of the phylogenetic groups was carried out using the PCRs described by Clermont et al. [15], which consists of a quadruplex PCR and two simple PCRs. This reaction is based on the amplification of three genes (*chuA*, *yjaA* and *arpA*) and a DNA fragment (TspE4.C2) that allows each of the strains to be classified in one of the eight phylogenetic groups of *E. coli* (A, B1, B2, C, D, E, F and *Escherichia clade* I).

The different PCR reactions were carried out in a final volume of 25 μL. Firstly, the quadruplex PCR was performed using the following quantities: 2 μL of DNA, 2.5 μL of 10X buffer (Bioline, London, UK), 2.5 μL of dNTPs (Bioline, London, UK), 1 μL of 50 mM MgCl_2_ (Bioline, London, UK), 2 μL of primers chuA.1b, chuA.2, yjaA.1b, yjaA.2b, TspE4.C2.1b, TspE4.C2.2b, AceK.f and ArpA1.R (Sigma Aldrich, Madrid, Spain), 1.5 U Immolase ™ DNA polymerase (Bioline, London, UK) and type II water (Millipore, Massachusetts, United States) to complete the final volume). The conditions of the reaction were the following: 4 min at 95 °C, followed by 30 cycles of 35 s at 95 °C, 45 s at 59 °C, 2 min at 72 °C and a final cycle of 5 min at 72°C. From the quadruple genotype obtained, either each strain was directly assigned to a phylogroup or two additional simple PCRs were performed by adding the specific primers E and C and their corresponding annealing temperatures (57 °C and 59 °C, respectively). The amplification reaction conditions were as follows: 4 min at 95°C, followed by 30 cycles of 35 s at 95 °C, 45 s at 59 °C, 2 min at 72 °C, and a final cycle of 5 min at 72 °C and using the same concentrations of reagents as in the previous case. After amplification, PCR products were separated by electrophoresis on 1% agarose gel in 1xTBE buffer, stained with ethidium bromide and visualized by UV transillumination. The molecular weight marker 1 kb plus DNA Ladder was used (Invitrogen, Barcelona, Spain).

### 4.4. Multilocus Sequence Typing (MLST)

To determinate the clonal dissemination of ESBL-producing *E. coli*, an ST analysis was performed following the scheme described by Wirth et al. [18]. Seven housekeeping genes selected from the MLST database of the University of Warwick were amplified and sequenced for each isolate. These genes code for the following proteins: adenylate cyclase (*adk*), fumarate hydratase (*fumC*), DNA gyrase (*gyrB*), isocitrate/isopropylmalate dehydrogenase (*icd*), malate dehydrogenase (*mdh*), adenylosuccinate (*purA*) and ATP/GTP binding motif (*recA*). The PCR reaction was performed according to the procedure described by Tartof et al. [57] in a total volume of 50 μL:3 μL of the previously extracted DNA, 5 μL of 10X buffer (Bioline, London, UK), 5 μL of dNTPs (Bioline, London, UK), 1.5 μL of 50 mM MgCl2 (Bioline, London, UK), 2 μL of each primer (Sigma Aldrich, Madrid, Spain), 1.5 U of Inmolase™ (Bioline, London, UK) and type II water (Millipore, Massachusetts, United States) to final volumen. The amplification reaction conditions were as follows: 3 min at 94 °C, followed by 30 cycles of 1 min at 59 °C, 1 min at the binding temperature of each primer, 2 min at 72 °C, and a final cycle of 5 min at 72 °C.

Subsequently, the amplicons were sequenced at the EZ-Seq service of Macrogen Spain and sequence data were imported into the *E. coli* MLST database website (http://mlst.warwick.ac.uk/mlst/dbs/Ecoli, URL accessed on 15 September 2022) to determine MLST type. Finally, the data for each allele, ST and CC were analyzed into the BioNumerics version 7.6 software (Applied Maths NV/bioMérieux, Sint-Martens-Latem, Belgium) and Minimum Spanning Tree (MST) were created based on the number of common alleles of each strain. This mathematical algorithm allows us to reconstruct from the MLST data the existing phylogenetic relationships between the different isolated strains and the different clonal groups. These relationships are represented in Figure 3 and Figure 4 by the connecting lines between the nodes (bold, solid, dashed or dotted lines according to the presence of 1, 2, 3 and 4 discordant alleles, respectively).

### 4.5. Statistical Analysis

The results were subjected to statistical processing with the Statistical Package for Social Sciences (SPSS) 15.0 software (SPSS Inc., Chicago, IL, USA), applying the Chi-square test (X2) with a significance level of *p* < 0.05.

## Data Availability

Not applicable.

## Supplementary Materials

The following supporting information can be downloaded at: https://www.mdpi.com/article/10.3390/antibiotics11111465/s1, Table S1: Main characteristics of the strains selected for this study.
