# Supplementary material for: Clonal Complexes 23, 10, 131 and 38 as Genetic Markers of the Environmental Spread of Extended-Spectrum β-Lactamase (ESBL)-Producing E. coli
Source: Antibiotics (Basel). 2022 Oct 24;11(11):1465. doi: 10.3390/antibiotics11111465 (PMC9686695; doi:10.3390/antibiotics11111465)
Supplement: Supplementary file 1 [file antibiotics-11-01465-s001.zip › antibiotics-1973701-supplementary.pdf]

SUPPLEMENTARY MATERIAL

Table S1. Main characteristics of the strains selected for this study

Nº strain	Origin[1]	Resistance gene	Phylogroup	ST	CC[2]	Antibiotic resistance profile[3]										
						Penicillins	Cephalosporins	Monobactams	β-lactamase inhibitors	Carbapenems	Aminoglycosides	Quinolones	Tetracyclines	Others	MDR	Extended MDR
1	Chicken (F)	SHV-12	A	ST6648	NA	AMP, PIP, MZ	CZ, CXM, CPD, CTX, CAZ, FEP	AZT	-	-	-	LV, CIP, MXF, NOR	TET	CHL	+	+
2	Chicken (H)	CTX-M-1	F	ST117	NA	AMP, PIP, MZ	CZ, CXM, CPD, CTX, CAZ, FEP	AZT	AMS	-	-	-	TET	SXT	+	+
3	Chicken (H)	SHV-12, TEM-171	B1	ST101	CC101	AMP, PIP, MZ	CZ, CXM, CPD, CTX, CAZ, FEP	AZT	-	-	-	LV, CIP, MXF, NOR	TET	CHL	+	+
4	Chicken (H)	CTX-M-1	C	ST23	CC23	AMP, PIP, MZ	CZ, CXM, CPD, CTX, CAZ, FEP	AZT	AMC, AMS, TZP	ETP, MER, IMP	-	LV, CIP, MXF, NOR	TET	SXT, FOT	+	+
5	Chicken (N)	SHV-12	F	ST2085	NA	AMP, PIP, MZ	CZ, CXM, CPD, CTX, CAZ, FEP	AZT	-	-	-	-	TET	CHL	+	+
6	Chicken (H)	SHV-12, TEML-278	Unknown	ST2599	NA	AMP, PIP, MZ	CZ, CXM, CPD, CTX, CAZ, FEP	AZT	-	-	-	LV, CIP, MXF, NOR	TET	SXT, CHL	+	+
7	Chicken (H)	CTX-M-1	A	ST373	CC168	AMP, PIP, MZ	CZ, CXM, CPD, CTX, CAZ, FEP	AZT	-	-	-	-	-	-	+	-
8	Chicken (H)	SHV-12, TEM-171	Unknown	ST2062	NA	AMP, PIP, MZ	CZ, CXM, CPD, CTX, CAZ, FEP	AZT	AMS	-	GM, TO	-	TET	CHL	+	+
9	Chicken (H)	SHV-12	F	ST354	CC354	AMP, PIP, MZ	CZ, CXM, CPD, CTX, CAZ, FEP	AZT	-	-	-	LV, CIP, MXF, NOR	TET	-	+	+
10	Turkey (M)	SHV-12	Clade I or II	ST23	CC23	AMP, PIP, MZ	CZ, CXM, CPD, CTX, CAZ, FEP	AZT	-	-	-	MXF	TET	CHL	+	+
11	Turkey (M)	SHV-12	A	ST unknown	NA	AMP, PIP, MZ	CZ, CXM, CPD, CTX, CAZ, FEP	AZT	-	-	-	LV, CIP, MXF, NOR	TET	CHL	+	+
12	Chicken (N)	SHV-12	A	ST665	NA	AMP, PIP, MZ	CZ, CXM, CPD, CTX, CAZ, FEP	AZT	-	-	-	MXF	TET	CHL	+	+
13	Chicken (S)	SHV-12, TEM-171	F	ST354	CC354	AMP, PIP, MZ	CZ, CXM, CPD, CTX, CAZ, FEP	AZT	-	-	-	LV, CIP, MXF, NOR	TET	SXT, CHL	+	+
14	Chicken (S)	SHV-12, CTX-M-1	A	ST93	CC168	AMP, PIP, MZ	CZ, CXM, CPD, CTX, CAZ, FEP	AZT	-	-	-	LV, CIP, MXF, NOR	TET	CHL	+	+
15	Chicken (F)	SHV-12, TEM-171	B1	ST937	NA	AMP, PIP, MZ	CZ, CXM, CPD, CTX, CAZ, FEP	AZT	-	-	-	-	-	-	+	-
16	Chicken (F)	SHV-12, TEM-171, CTX-M-1	A	ST10	CC10	AMP, PIP, MZ	CZ, CXM, CPD, CTX, CAZ, FOX, FEP	AZT	AMC	-	GM, TO	-	-	-	+	+
17	Chicken (F)	SHV-12	F	ST117	NA	AMP, PIP, MZ	CZ, CXM, CPD, CTX, CAZ, FEP	AZT	-	-	-	-	TET	-	+	-
18	Chicken (F)	CTX-M14	F	ST117	NA	AMP, PIP, MZ	CZ, CXM, CPD, CTX, CAZ, FEP	AZT	AMS	-	-	LV, CIP, MXF, NOR	TET	SXT	+	+
19	Chicken (N)	-	B1	ST1524	NA	AMP, PIP, MZ	CZ, CXM, CPD, CTX, CAZ, FEP	AZT	AMS	-	-	LV, CIP, MXF, NOR	TET	CHL	+	+
20	Chicken (F)	SHV-12, TEM-171, CTX-M-14	A	ST6094	NA	AMP, PIP, MZ	CZ, CXM, CPD, CTX, CAZ, FEP	AZT	-	-	-	LV, CIP, MXF, NOR	-	-	+	-
21	Turkey (F)	TEML-278	B1	ST unknown	NA	AMP, PIP, MZ	CZ, CXM, CPD, CTX, CAZ, FOX, FEP	AZT	AMS	-	-	CIP, MXF	TET, TIG	SXT	+	+
22	Water (R)	TEML-278, CTX-M-14	B1	ST1434	NA	AMP, PIP, MZ	CZ, CXM, CPD, CTX, CAZ, FOX, FEP	AZT	AMS	-	GM, TO	LV, CIP, MXF, NOR	TET	SXT, FOT, CHL	+	+
23	Water (R)	TEML-278, CTX-M-14	B1	ST1434	NA	AMP, PIP, MZ	CZ, CXM, CPD, CTX, FOX, FEP	AZT	AMC, AMS	-	GM, TO	LV, CIP, MXF, NOR	TET	SXT, FOT, CHL	+	+
24	Water (R)	TEML-278, CTX-M-14	A	ST746	NA	AMP, PIP, MZ	CZ, CXM, CPD, CTX, FOX, FEP	AZT	AMC, AMS	-	GM, TO	LV, CIP, MXF, NOR	TET	SXT, FOT, CHL	+	+
25	Water (R)	CTX-M-15	C	ST410	CC23	AMP, PIP, MZ	CZ, CXM, CPD, CTX, CAZ, FOX,FEP	AZT	AMC, AMS	MER	-	LV, CIP, MXF, NOR	TET, TIG	SXT, CHL	+	+
26	Water (R)	TEML-278, CTX-M-14	B1	ST2599	NA	AMP, PIP, MZ	CZ, CXM, CPD, CTX, CAZ, FEP	AZT	AMS	-	-	LV, CIP, MXF, NOR	TET	-	+	+
27	Water (R)	CTX-M-1	A	ST1486	NA	AMP, PIP, MZ	CZ, CXM, CPD, CTX, CAZ, FEP	AZT	-	-	-	-	TET	CHL	+	+
28	Water (R)	CTX-M-1	A	ST1486	NA	AMP, PIP, MZ	CZ, CXM, CPD, CTX, CAZ, FEP	AZT	-	-	-	-	TET	CHL	+	+
29	Water (R)	TEML-278, SHV-12	B1	ST971	NA	AMP, PIP, MZ	CZ, CXM, CPD, CTX, CAZ, FEP	AZT	-	-	-	-	TET		+	-
30	Water (R)	-	Unknown	ST unknown	NA	-	-	-	-	-	-	-	-	COL	-	-
31	Water (W)	CTX-M-1	A	ST6701	NA	AMP, PIP, MZ	CZ, CXM, CPD, CTX, CAZ, FEP	AZT	AMS	-	-	-	TET	-	+	+
32	Water (W)	TEML-278, CTX-M-14	B2	ST131	CC131	AMP, PIP, MZ	CZ, CXM, CPD, CTX, CAZ, FEP	-	-	-	-	LV, CIP, MXF, NOR	-	-	+	-
33	Water (W)	CTX-M-14	D	ST2914	CC349	AMP, PIP, MZ	CZ, CXM, CPD, CTX, CAZ, FOX, FEP	AZT	AMS	-	-	-	-	SXT, FOT	+	+

Nº strain	Origin[1]	Resistance gene	Phylogroup	ST	CC[2]	Antibiotic resistance profile[3]									MDR	Extended MDR
						Penicillins	Cephalosporins	Monobactams	β-lactamase inhibitors	Carbapenems	Aminoglycosides	Quinolones	Tetracyclines	Others		
34	Water (W)	CTX-M-15	B2	ST636	NA	AMP, PIP, MZ	CZ, CXM, CPD, CTX, CAZ, FEP	AZT	-	-	-	-	-	SXT	+	-
35	Water (W)	SHV-12, CTX-M-1	A	ST10	CC10	AMP, PIP, MZ	CZ, CXM, CPD, CTX, CAZ, FEP	AZT	-	-	-	LV, CIP, MXF, NOR	-	CHL	+	+
36	Water (W)	TEML-278, SHV-12, CTX-M-1	B1	ST162	CC469	AMP, PIP, MZ	CZ, CXM, CPD, CTX, CAZ, FEP	AZT	AMC, AMS, TZP	-	-	LV, CIP, MXF, NOR	TET	SXT, CHL	+	+
37	Water (W)	TEM-171, CTX-M-1	B1	ST1123	NA	AMP, PIP, MZ	CZ, CXM, CPD, CTX, CAZ, FEP	AZT	AMS	-	-	-	-	-	+	-
38	Water (W)	TEML-278, CTX-M-14	D	ST38	CC38	AMP, PIP, MZ	CZ, CXM, CPD, CTX, CAZ, FEP	-	AMS	-	GM, TO	-	TET	SXT	+	+
39	Water (W)	TEML-278, CTX-M-14	D	ST38	CC38	AMP, PIP, MZ	CZ, CXM, CPD, CTX, CAZ, FEP	-	AMS	-	GM, TO	-	TET	SXT	+	+
40	Water (W)	TEML-278, CTX-M-1	D	ST unknown	NA	AMP, PIP, MZ	CZ, CXM, CPD, CTX, CAZ, FEP	AZT	-	-	GM, TO	LV, CIP, MXF, NOR	TET	SXT, FOT	+	+
41	Water (W)	TEM-171, CTX-M-15	A	ST4599	NA	AMP, PIP, MZ	CZ, CXM, CPD, CTX, CAZ, FEP	AZT	-	-	-	-	TET		+	-
42	Water (W)	TEML-278, CTX-M-15	A	ST227	CC10	AMP, PIP, MZ	CZ, CXM, CPD, CTX, CAZ, FEP	AZT	AMC, AMS	-	-	-	-	FOT	+	+
43	Water (W)	TEM-176, CTX-M-14, CTX-M-15	E	ST130	CC31	AMP, PIP, MZ	CZ, CXM, CPD, CTX, CAZ, FEP	-	-	-	-	-	-		-	-
44	Water (W)	OXA-1, CTX-M-15	B2	ST131	CC131	AMP, PIP, MZ	CZ, CXM, CPD, CTX, CAZ, FEP	AZT	AMC, AMS	-	TO	LV, CIP, MXF, NOR	-	SXT	+	+
45	Water (W)	TEML-278	A	ST52	NA	AMP, PIP, MZ	CZ, CXM, CPD, CTX, FOX, FEP	AZT	AMC, AMS, TZP	ETP, MER, IMP	-	LV, CIP, MXF, NOR	-	SXT, FOT	+	+
46	Water (C)	CTX-M-15	B1	ST297	NA	AMP, PIP, MZ	CZ, CXM, CPD, CTX, CAZ, FEP	AZT	AMC, AMS	-	-	LV, CIP, MXF, NOR	TET	SXT, CHL	+	+
47	Water (C)	CTX-M-15	Unknown	ST unknown	NA	AMP, PIP, MZ	CZ, CXM, CPD, CTX, CAZ, FOX, FEP	AZT	AMC, AMS, TZP	-	-	-	-	COL, FOT	+	+
48	Water (C)	*mcr-1*	B2	ST155	CC155	-	-	-	-	-	-	LV, CIP, MXF, NOR	TET	COL, SXT	+	-
49	Healthy carrier	TEM-116, CTX-M14	B1	ST971	NA	AMP	CZ, CXM, FEP	-	-	-	-	-	TET	COL	+	-
50	Healthy carrier	TEM-116, CTX-M14	A	ST2404	NA	AMP, PIP, MZ	CZ, CXM, CPD, CTX, CAZ, FEP	AZT	-	-	GM, TO	-	TET	STX	+	+
51	Healthy carrier	TEML-171, CTX-M1	D	ST38	CC38	AMP, MZ	CZ, CXM, CPD, CAZ, FOX	-	AMC, AMS	-	-	-	-	-	+	-
52	Healthy carrier	TEML-171, CTX-M1	Unknown	ST unknown	NA	AMP, PIP, MZ	CZ, CXM, CPD, CTX, CAZ, FEP	AZT	AMS	-	-	-	-	STX	+	+
53	Healthy carrier	TEM-171, CTX-M14, CTX-M15	B2	ST131	CC131	AMP, PIP, MZ	CZ, CXM, CPD, CTX, CAZ, FEP	AZT	AMS	-	-	-	-	STX	+	+
54	Healthy carrier	CTX-M14	A	ST unknown	NA	AMP, PIP, MZ	CZ, CXM, CPD, CTX, CAZ, FEP	AZT	-	-	-	-	TET	STX	+	+
55	Healthy carrier	TEM-171	A	ST unknown	NA	AMP, PIP, MZ	CZ, CXM, CPD, CTX, CAZ, FEP	AZT	-	-	-	LV, CIP, MXF, NOR	TET	STX	+	+
56	Healthy carrier	TEM-171, CTX-M15	B2	ST131	CC131	AMP, PIP, MZ	CZ, CXM, CPD, CTX, CAZ, FEP	AZT	AMC, AMS	-	-	LV, CIP, MXF, NOR	-	COL	+	+
57	Healthy carrier	TEM-171, CTX-M15	B2	ST131	CC131	AMP, PIP, MZ	CZ, CXM, CPD, CTX, CAZ, FEP	AZT	AMS	-	-	LV, CIP, MXF, NOR	-	FOT	+	+
58	Healthy carrier	CTX-M14	B2	ST131	CC131	AMP, PIP, MZ	CZ, CXM, CPD, CTX, CAZ, FEP	AZT	-	-	-	LV, CIP, MXF, NOR	-	-	+	-
59	Healthy carrier	CTX-M14	B2	ST131	CC131	AMP, PIP, MZ	CZ, CXM, CPD, CTX, CAZ, FEP	AZT	-	-	-	LV, CIP, MXF, NOR	-	-	+	-
60	Healthy carrier	CTX-M15	C	ST unknown	NA	AMP, PIP, MZ	CZ, CXM, CPD, CTX, CAZ, FEP	AZT	AMS	-	-	LV, CIP, MXF, NOR	-	STX	+	+
61	Healthy carrier	-	D	ST unknown	NA	AMP, PIP, MZ	CZ, CXM, CPD, CTX, CAZ, FEP	AZT	-	-	-	LV, CIP, MXF, NOR	-	STX	+	+

[1] F, fillet; H, hamburger; N, nugget; M, meatballs; S, sausage; R, river; W, WWTP; C, collector.

[2] NA: not associated with any known clonal complex.

[3]AMP, ampicillin; PIP, piperacillin; MZ, mezlocillin; CZ, cefazolin; CXM, cefuroxime; CPD, cefpodoxime; CTX, cefotaxime; CAZ, ceftazidime; FOX, cefoxitin; FEP, cefepime; AZT, aztreonam; AMC, amoxicillin-clavulanic acid; AMS, ampicillin-sulbactam; TZP, piperacillin-tazobactam; ETP, ertapenem; MER, meropenem; IMP, imipenem; GM, gentamicin; TO, tobramycin; LV, levofloxacin; CIP, ciprofloxacin; MXF, moxifloxacin; NOR, norfloxacin; TET, tetracycline; TIG, tigecycline; SXT, trimethoprim-sulfamethoxazole; COL, colistin; FOT, fosfomycin; CHL, chloramphenicol.
